# Effects of Visual Working Memory on Brain Information Processing of Irrelevant Auditory Stimuli

**DOI:** 10.1371/journal.pone.0089989

**Published:** 2014-02-26

**Authors:** Jiagui Qu, Joshua D. Rizak, Lun Zhao, Minghong Li, Yuanye Ma

**Affiliations:** 1 University of Science and Technology of China, Hefei, Anhui, P. R. China; 2 State Key Laboratory of Brain and Cognitive Science, Kunming Institute of Zoology, Chinese Academy of Sciences, Kunming, Yunnan, P. R. China; 3 University of the Chinese Academy of Science, Beijing, P. R. China; 4 Psychological Research Center, Beijing Yiran Sunny Technology Co. Lt, Beijing, P. R. China; 5 State Key Laboratory of Brain and Cognitive Science, Institute of Biophysics, Chinese Academy of Sciences, Beijing, P. R. China; Goldsmiths, University of London, UK, United Kingdom

## Abstract

Selective attention has traditionally been viewed as a sensory processing modulator that promotes cognitive processing efficiency by favoring relevant stimuli while inhibiting irrelevant stimuli. However, the cross-modal processing of irrelevant information during working memory (WM) has been rarely investigated. In this study, the modulation of irrelevant auditory information by the brain during a visual WM task was investigated. The N100 auditory evoked potential (N100-AEP) following an auditory click was used to evaluate the selective attention to auditory stimulus during WM processing and at rest. N100-AEP amplitudes were found to be significantly affected in the left-prefrontal, mid-prefrontal, right-prefrontal, left-frontal, and mid-frontal regions while performing a high WM load task. In contrast, no significant differences were found between N100-AEP amplitudes in WM states and rest states under a low WM load task in all recorded brain regions. Furthermore, no differences were found between the time latencies of N100-AEP troughs in WM states and rest states while performing either the high or low WM load task. These findings suggested that the prefrontal cortex (PFC) may integrate information from different sensory channels to protect perceptual integrity during cognitive processing.

## Introduction

Working memory (WM) and selective attention (SA) are two key components of cognitive processing that are intimately related [1], [2], [3]. WM is a specific type of short-term memory that enables the brain to temporarily maintain specific information as well as process this information to direct future behaviors [2]. Therefore, WM is considered the basic neural mechanism required to accomplish executive functions [1]. SA is a cognitive process that filters incoming information to maintain ongoing cognition [3]. By filtering incoming information, SA allows only relevant information into the short-term processing stores of WM [4], [5]. This important cognitive relationship allows an organism to focus attention on external stimuli, tasks and challenges, maintain necessary information temporarily and to make appropriate behaviors according to the purpose or challenge. WM also has a regulatory function on selective attention processes [5]. It has been shown that a higher WM load in a visual WM task results in greater interference from distractor faces on behavioral performance [6]. Likewise, distractor interference in an auditory task has been found to increase significantly under high (vs. low) loads in a concurrent WM task compared with that in an inconcurrent WM task [7]. These results suggested that the regulation of selective attention by WM depended on the level and type of the WM load.

The prefrontal cortex (PFC) is directly involved in the modulation of irrelevant stimuli [8], [9], [10] and is the key brain region utilized in WM and selective attention processes [11], [12], [13], [14]. The activity of the PFC has been found to be significantly increased during WM tasks [15]. Although much has been learned from these studies, the majority of these studies were concerned with the relationship of stimuli received within the same sensory channel, and little is known about how or if the brain can modulate irrelevant stimuli from different sensory sources, i.e., visual versus auditory systems, in a cross-modal manner. To evaluate whether the PFC and/or other brain regions are able to modulate irrelevant stimuli from different sensory channels in a cross-modal manner, a delayed-response task (DRT) that involved visual WM was employed along with an irrelevant auditory stimulus. In this task, the auditory stimulus was applied to determine whether the brain could maintain attention toward the visual WM task while dampening and/or inhibiting the response to the auditory stimulus. Electroencephalography (EEG) was used to monitor brain activity during the visual WM task. In the EEG, the amplitudes of auditory evoked potentials (AEP) are positively related to the cognitive activities of brain regions [16], [17], [18]. The AEP is composed of three main components (P50, N100, and P200), which are known as the middle latency AEP. The N100-AEP has been related to the trigger of attention [19], [20] and was used in the present study as an indicator of brain activity following irrelevant auditory stimuli in a cross-modal system.

In this cognitive experiment, human subjects were asked to perform a visual object WM task with either a low or high WM load. An irrelevant auditory stimulus was presented in each delayed phase between object presentation and object recognition and in each inter-trial interval (ITI) phase. The N100-AEP was recorded to monitor brain activity associated with the irrelevant auditory stimulus in the following regions of interest (ROI): the prefrontal, frontal, and temporal regions.

## Materials and Methods

### Ethics Statement

The research protocol of the study entitled “Effects of Visual working memory load on brain information processing of irrelevant auditory stimuli” has been reviewed and approved by the internal review board of Kunming Institute of Zoology, Chinese Academy of Sciences. Written informed consent has been obtained from the participants and the data was analyzed anonymously.

### Participants

Participants were undergraduate and graduate students randomly selected from colleges and universities in Kunming, China. The participants were between 19 and 27 years of age and the sex ratio was 1 to 1. Participants were divided into 2 groups in order to assess the effect of different WM loads. A total of 12 participants were assigned to the low WM load task (6 men, 6 women, mean age = 23); however, data from only 11 participants were analyzed due to technical problems with the EEG apparatus during the collection of 1 dataset. A total of 14 participants were assigned to the high WM load task (7 men, 7 women, mean age = 24). All participants were right-handed and had normal audition, normal vision, no history of neurological disease or drug addiction. The participants were asked to refrain from smoking and drinking tea, coffee, or alcohol for 8 hours prior to the memory test.

First, the participants were requested to read the experimental instructions and perform 5 test trials to become familiar with the task. In the formal experiments, the participants were asked to focus on a fixation rectangle and to ignore the sound clicks. The percentage of correct responses in the visual WM task was required to be higher than 85%. No subjects were removed from the study due to failure to meet this requirement.

### Memory Task Description

The delayed-response task (DRT) is a classic paradigm used to research the mechanisms of information maintenance and processing in the brain [21]. The DRT is composed of four phases: (1) target phase, in which target stimuli are presented to the participants; (2) delayed phase, in which the target stimuli are removed for delayed intervals of 3000–5000 ms; (3) probe phase, in which probe stimuli are presented to the participants and the participants are asked to identify whether the probe stimuli are the same as the target stimuli; and (4) inter-trial interval (ITI) phase, in which the participants are given a rest period of 5000–7000 ms prior to being presented with a new four phase trial. This paradigm is designed to evaluate information encoding, preservation, extraction, and recall.

### Experiment Design

Experiments were conducted with two WM loads (low and high visual WM load) because the accuracy of WM tasks with higher loads was found to be lower than 85% in preliminary tests.

#### 1. Low visual WM load task

At the beginning of each trial, a rectangle and a fixation point were presented for 500 ms to alert the participant to the task. Then, a black and white image of one Caucasian face was presented as the target stimulus for 1500 ms (target phase) with a visual angle of 3.5° horizontally and 5.5° vertically. After that, a delayed phase was randomly selected with a time interval between 3000∼5000 ms. Then another face was presented for 1000 ms and the participant was given 1000 ms (probe phase) to make a judgment whether the face was the same as the face presented in target phase. The image presented in the probe phase was the same image presented in the target phase in 50% of the trials. The order of correct and incorrect image pairings was randomized. After the judgment, there was a random time interval of 5000∼7000 ms (ITI phase) before the next trial began. Each task contained 80 trials.

#### 2. High visual WM load task

The high visual WM load task was performed with the same procedure as the low visual WM load task except that the visual object stimuli was a black and white image that contained two parallel Caucasian faces. The participant was asked to identify whether both of the faces presented in probe phase were the same as the faces presented in the target phase. Each task also contained 80 trials.

In both memory tasks, an external loudspeaker presented one irrelevant auditory stimulus at the midpoint of the delayed phase (WM state) and ITI phase (rest state) of each trial. The auditory stimuli were clicks compiled by Matlab from a series of sine waves generated for 10 ms. The click frequency was 1000 Hz and the click intensity was 85 dB.

Visual WM tasks were presented on a 17-inch computer screen with a pure grey background, a resolution of 1024-by-768 pixels and a refresh rate of 75 Hz. The distance between the eyes of the participants and the screen was 57 cm. The test was performed in a dark, sound-dampened room to eliminate any uncontrolled external stimuli. The visual objects presented as stimuli (for both the low and high WM load tasks) consisted of 150 black-and-white photos of young Caucasian male and female faces and the sex ratio of the faces was 1∶1. The brightness and contrast of the photos were constant with the background.

### Data Recording and Analysis

Raw EEG data were recorded and collected by a 64-leads Neuroscan EEG workstation. The AC filter was set at 0.1∼100 Hz and the data sampling rate was 1000 Hz. The reference electrode was positioned on the apex of the nose and the electric resistances of all electrodes were lower than 5 kΩ. Off-line data were analyzed with the data analysis software “Scan 4.3” to remove refused blocks and to reduce vertical ocular artifacts. The epoch data consisted of data segments from 100 ms before the triggers to 500 ms after the triggers. The data recorded at 100 ms before the trigger was applied as a baseline to normalize the data. The data was averaged by applying a low-pass filtering at 30 Hz (12 db). Following this step, N100-AEP troughs were automatically detected by the Neuroscan software. The identification standard of the N100-AEP was designated as the most significant trough between 75 ms∼175 ms and the latency of N100-AEP components were defined by the trough apex. The amplitudes of the N100-AEP were measured using the mean amplitudes from 10 ms before the trough to 10 ms after the trough according to the overall average troughs [22]. This study focused on both the changes of mean amplitudes and latencies of the N100-AEP. According to the nearby electrode similarity principle, we combined several ROIs into one ROI by pooling nearby electrodes together. These pooled ROIs include the left-prefrontal region (FP1 and AF3), mid-prefrontal region (FPZ), right-prefrontal region (FP2 and AF4), left-frontal region (F1, F3, F5, F7, FC1, FC3 and FC5), mid- frontal region (FZ and FCZ), right-frontal region (F2, F4, F6, F8, FC2, FC4 and FC6), left-temporal region (FT7, T7 and TP7), and right-temporal region (FT8, T8 and TP8), respectively. Each ROI consisted of the averaged value of the pooled electrodes.

All data were then processed with the SPSS 17.0 software package for statistical analysis. A repeated measures two-way ANOVA for state (WM vs rest) and load (low vs high) was performed for each ROI. The WM state and rest state N100-AEP amplitudes for each region were then set as two levels of the within-subject factor for the repeated measures one-way ANOVA. Each WM group (low or high) were treated separately. The latency data of the N100-AEP failed to meet the qualification standards of a repeated measures one-way ANOVA. Therefore, nonparametric statistics (Wilcoxon paired test) were applied to identify within-group relationships for the latencies between the WM state and the rest state. A statistically significant standard was set at *P*<0.05 with a 95% confidence interval.

## Results

Participants were evaluated with an EEG to measure the amplitudes of the N100-AEP following an auditory click during visual WM task. All participants complied with an accuracy requirement to complete each task with a correct response rate greater than 85% in both the low (97.33%) and high (94.17%) load WM tasks (Table 1).

**Table 1:** The percentage of correct responses in the visual WM tasks.

	The percentage of correct responses (%)
Low WM load	97.33 (2.85)
High WM load	94.17 (7.62)

The percentage of correct responses is greater than 85% in both the low and high WM load tasks. Data is shown as mean (SD).

**Figure 1:**
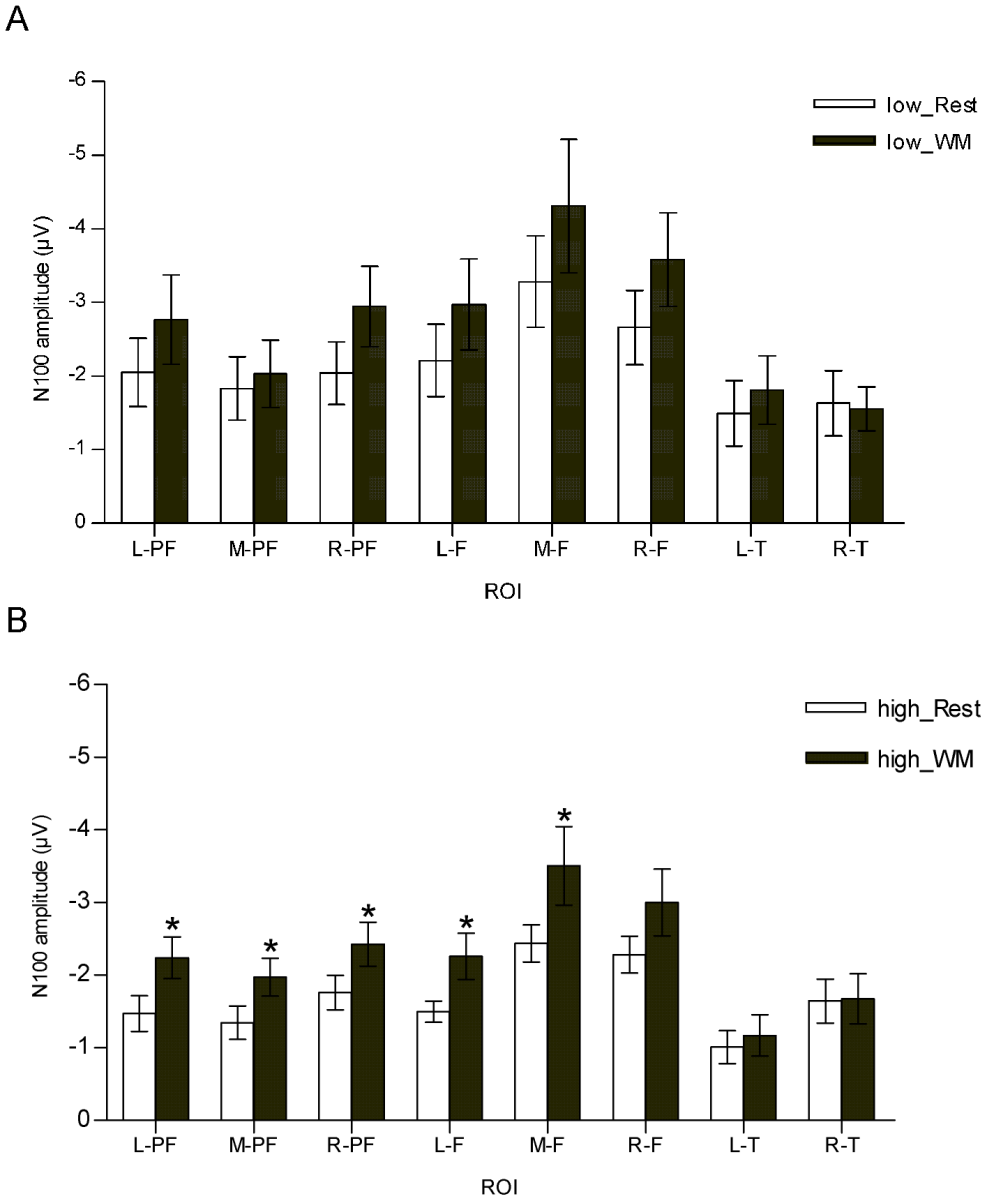
Mean N100-AEP amplitudes in rest states (white bars) and WM states (gray bars) following an irrelevant auditory stimulus. A: N100-AEP amplitudes in low WM load task; B: N100-AEP amplitudes in High WM load task. L-PF: left-prefrontal; M-PF: mid-prefrontal; R-PF: right-prefrontal; L-F: left-frontal; M-F: mid-frontal; R-F: right-frontal; L-T: left-temporal; R-T: right-temporal. Results are expressed as mean±SEM; *P<0.05.

**Figure 2:**
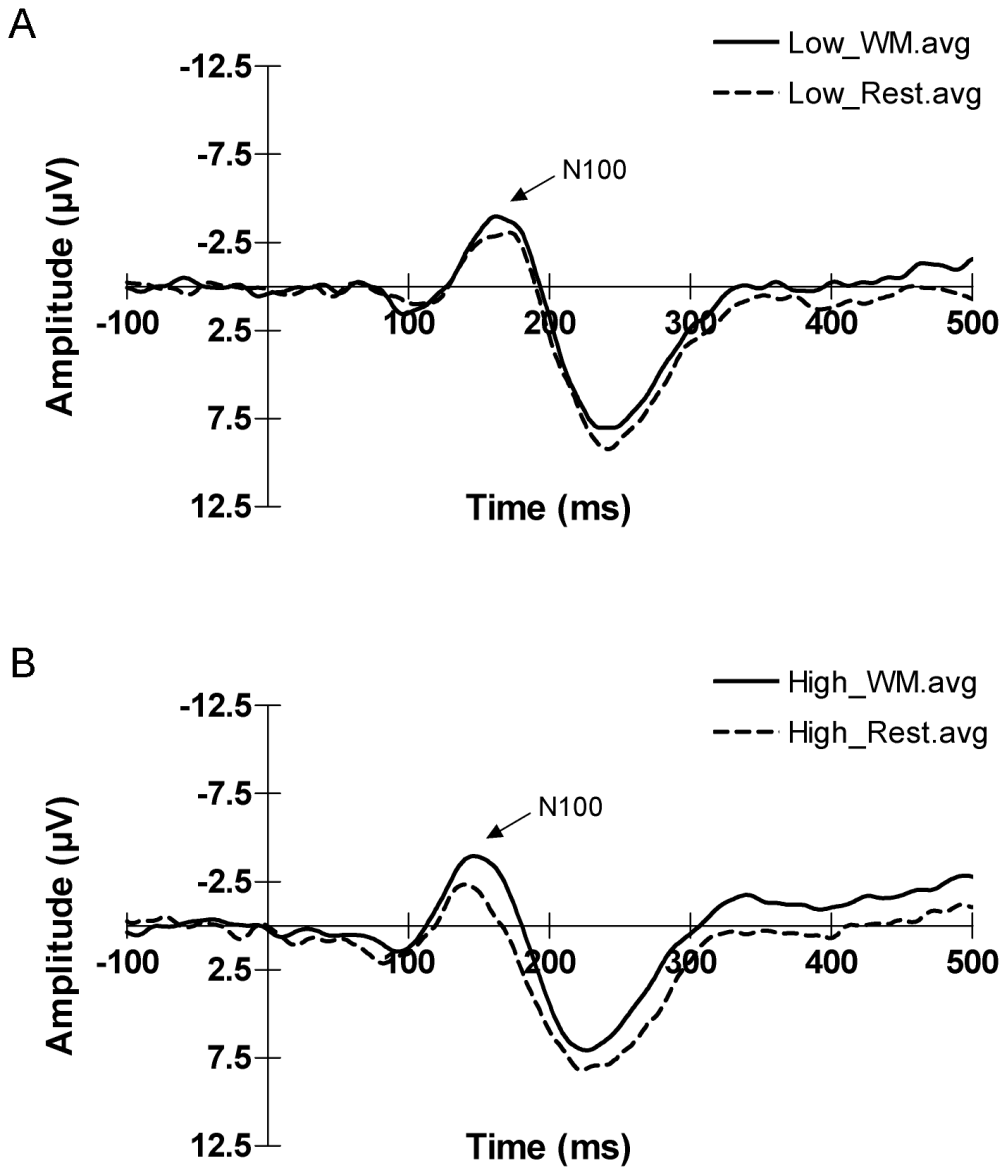
Overall average N100-AEP waveforms of the mid-frontal region. A: Waveforms of the low WM load task; B: Waveforms of the high WM load task. Solid lines represent the average N100-AEP waveform in the WM state; dotted lines represent the average N100-AEP waveform in the rest state.

A repeated measures two-way ANOVA for state (WM vs rest) and load (low vs high) revealed no interaction between state and load in all ROIs [L-PF, M-PF, R-PF, L-F, M-F, R-F, L-T and R-T (F_(1, 23)_ = 0.008, 1.168, 0.250, 0.000, 0.002, 0.086, 0.083, 0.040), P>0.05]. Whereas there was significant differences for N100-AEP amplitudes between WM states and rest states in six ROIs [L-PF, M-PF, R-PF, L-F, M-F and R-F (F_(1, 23)_ = 6.846, 4.389, 10.544, 5.991, 6.505 and 5.599), *P*<0.05]. In the low WM load task paradigm, a one-way ANOVA comparison of N100-AEP amplitudes in WM states (Delay Phase) and rest states (ITI Phase) revealed no statistically significant differences in all ROIs [left-prefrontal, mid-prefrontal, right-prefrontal, left-frontal, mid-frontal, right-frontal, left-temporal and right-temporal (F_(1, 10)_ = 1.557, 0.339, 4.025, 1.676, 1.808, 2.176, 0.617 and 0.035, *P*>0.05), Figure 1A]. However, the one-way ANOVA comparisons of N100-AEP amplitudes in the high WM load task were found to be significantly affected during WM processing in 5 ROIs: the left-prefrontal, mid-prefrontal, right-prefrontal, left-frontal, and mid-frontal [(F_(1,13)_ = 11.563, 7.885, 7.505, 6.248 and 6.650, *P*<0.05, Figure 1B]. It is noteworthy that the N100-AEP amplitudes in WM states were higher (although not statistically significant) than that in rest states in both low and high WM load tasks and that this trend was witnessed in all recorded brain regions. The overall average N100-AEP waveforms of the mid-frontal brain region for both the low and high WM load tasks are displayed in Figure 2.

The time latency of the largest trough of the N100-AEP following the auditory click was also evaluated. Application of Wilcoxon paired test to the N100-AEP latencies of WM states and rest states did not reveal any statistically significant difference (*P*>0.05) in all ROIs in either the low or high WM load tasks (Table 2 and 3).

**Table 2:** Time Latencies of the N100-AEP trough in each brain region in WM states and rest states in the low WM load tasks.

Brain Region	WM states (ms)	Rest states (ms)	*P* values of Wilicoxon test
L-PF	166.5(17.66)	163.37(20.96)	0.128
M-PF	165.09(18.35)	163.18(21.28)	1.000
R-PF	167.14(19.49)	163.23(21.63)	0.192
L-F	163.7(17.41)	160.42(20.14)	0.798
M-F	162.32(17.21)	162.18(20.34)	0.760
R-F	165.04(17.59)	163.57(21.35)	0.790
L-T	160.76(21.76)	157(22.03)	0.534
R-T	163.85(21.07)	156.64(25.61)	0.683

There were no statistically significant differences (*P*>0.05) in the N100-AEP latencies between WM states and rest states in the low load tasks. L-PF: left-prefrontal; M-PF: mid-prefrontal; R-PF: right-prefrontal; L-F: left-frontal; M-F: mid-frontal; R-F: right-frontal; L-T: left-temporal; R-T: right-temporal. Data is shown as mean (SD).

**Table 3:** Time Latencies of the N100-AEP trough in each brain region in WM states and rest states in the high WM load tasks.

WM states/high WM load (ms)	Rest states/high WM load (ms)	*P* values of Wilicoxon test
150.93(14.97)	144.54(15.94)	0.090
150.93(16.18)	144.64(16.33)	0.100
150.82(14.92)	146.64(11.69)	0.073
149.31(11.65)	144.52(11.3)	0.100
149.25(13.76)	146.43(11.73)	0.126
150.93(14.79)	148.22(10.14)	0.100
149.55(11.39)	148.81(11.05)	0.109
152.29(14.86)	153.55(13.48)	0.109

There were no statistically significant differences (*P*>0.05) in the N100-AEP latencies between WM states and rest states in the high load tasks. L-PF: left-prefrontal; M-PF: mid-prefrontal; R-PF: right-prefrontal; L-F: left-frontal; M-F: mid-frontal; R-F: right-frontal; L-T: left-temporal; R-T: right-temporal. Data is shown as mean (SD).

## Discussion

Previous research has suggested that selective attention processes in the PFC are able to inhibit irrelevant stimuli signals during WM tasks [23], [24]. However, these studies were performed within the same sensory channel. In the present study, a delayed-response task that involved visual WM and an irrelevant auditory stimulus was employed to study the role of the PFC in processing irrelevant stimuli from different sensory channels. Our results suggested that the processing of irrelevant auditory stimuli was affected during the visual WM task as the N100-AEP amplitudes induced by the irrelevant stimulus were larger in WM states than that in rest states. Furthermore, there were no differences in the N100-AEP latencies in the WM and rest states, which suggested that the visual and auditory stimuli might be processed in a parallel manner. It seems that the load of the WM task might also play a role in this process as comparison of N100-AEP amplitudes between WM states and rest states found significant differences only in the high load WM task, but not in the low load WM task. However, there was no interaction between state and load found in a two-way ANOVA, which makes the role of WM load uncertain. As two different groups of participants were used in this study to avoid a learning effect, the absence of an interaction might be related to an uneven baseline between two separate groups of participants. It is also possible that there was no interaction between state and load under the conditions of our study. Further research will be needed to clarify the role of WM load on cross-modal sensory processing.

The PFC has been regarded as an integrator of information from multiple sources. Pyramidal neurons in the PFC have the largest number of dendritic spines [25], which suggests that neurons in the PFC have the ability to integrate a large number of excitatory inputs. The ability to integrate a large number of excitatory inputs is very important for information integrating and processing. Our results showing that the N100-AEP amplitudes were affected in the visual WM tasks reflected integrated information processing of both visual and auditory channels by the PFC. This information processing mechanism might play an important role for humans to adapt to new environments and provide for better implementation of executive functions.

It is also noteworthy that the five brain regions (left-prefrontal, mid-prefrontal, right-prefrontal, left-frontal, and mid-frontal) with significant differences in the N100-AEP amplitudes overlap with regions that are closely related to object recognition and WM [12], [26], [27]. This suggested that the visual WM might participate in the modulation of irrelevant auditory stimuli.

Taken together, our results suggest that the PFC may integrate information from both visual and auditory channels and modulate WM and selective attention in a cross-modal manner. Our findings also indicated that the PFC might have to integrate as much information as possible from all sensory channels prior to performing executive functions in order to adapt to complex and changing environments.
